# Insights from qualitative research on NAFLD awareness with a cohort of T2DM patients: time to go public with insulin resistance?

**DOI:** 10.1186/s12889-020-09249-5

**Published:** 2020-07-20

**Authors:** Mireia Alemany-Pagès, Mariana Moura-Ramos, Sara Araújo, Maria Paula Macedo, Rogério T. Ribeiro, Dulce do Ó, João Ramalho-Santos, Anabela Marisa Azul

**Affiliations:** 1 CNC-Center for Neuroscience and Cell Biology, CIBB, Rua Larga, University of Coimbra, Rua Larga, 3000-504 Coimbra, Portugal; 2 University of Coimbra, IIIUC-Institute for Interdisciplinary Research, 3030-789 Coimbra, Portugal; 3 Centro Hospitalar e Universitário de Coimbra, Reproductive Medicine Unit, Praceta Prof. Mota Pinto, 3000-075 Coimbra, Portugal; 4 Center for Research in Neuropsychology and Cognitive Behavioral Intervention, Faculty of Psychology and Educational Sciences, University of Coimbra, Rua do Colégio Novo, 3000-115 Coimbra, Portugal; 5 CES-Centre for Social Studies, University of Coimbra, 3000-104 Coimbra, Portugal; 6 APDP-Portuguese Diabetic Association, 1250-189 Lisbon, Portugal; 7 CEDOC-Centro de Estudos de Doenças Crónicas, NOVA Medical School/Faculdade de Ciências Médicas, 1150-082 Lisbon, Portugal; 8 Department of Medical Sciences, University of Aveiro, 3810-193 Aveiro, Portugal; 9 Department of Life Sciences, Calçada Martim de Freitas, University of Coimbra, 3000-456 Coimbra, Portugal

**Keywords:** NAFLD, Awareness, Knowledge, Education, Self-management, T2DM, Qualitative research, Risk communication

## Abstract

**Background:**

It is estimated that around 70% of Type 2 Diabetes Mellitus patients (T2DM) have Non-Alcoholic Fatty Liver Disease (NAFLD). Awareness and education are amongst the major shortcomings of the public health response to the increasing threat of NAFLD. Characterizing the specific NAFLD-related information needs of particular high-risk metabolic communities, for instance, T2DM patients, might aid in the development of evidence-based health promotion strategies, ultimately promoting NAFLD-awareness, treatment adherence and therapeutic success rates.

**Methods:**

Semi-structured interviews with T2DM patients were conducted to gain insight into their awareness of NAFLD, including its relationship with insulin resistance and T2DM.

**Results:**

Awareness of NAFLD as a disease entity, as well as its progression to end-stage liver disease or its relationship with other metabolic conditions, including insulin resistance and T2DM was low. Surveillance behaviours were also suboptimal and perceptions on the self-management knowledge and praxis regarding lifestyle intervention components of T2DM treatment seemed detached from those of NAFLD.

**Conclusions:**

Our findings could inform the integration of NAFLD-related content in T2DM health promotion strategies. Rising awareness on NAFLD progression and its relationship with T2DM using culturally and community-relevant constructs might facilitate the development of primary and secondary prevention programmes to promote the adherence to lifestyle interventions by influencing NAFLD threat perceptions.

## Background

Non-Alcoholic Fatty Liver Disease (NAFLD), affecting 25% of the global population, is currently the most common chronic liver disease worldwide and constitutes an increasing threat to public health both at economic and clinical levels [1–3]. The most benign form of NAFLD is characterized by the presence of micro- or macro-vesicular steatosis in ≥5% of hepatocytes in the absence of other recognized aetiological factors [4]. However, the NAFLD continuum ranges from simple hepatic steatosis to the more severe form of Non-Alcoholic Steatohepatitis (NASH), a state of inflammation that in turn can progress to end-stage liver disease, including cirrhosis and Hepatocellular Carcinoma (HCC) [4–6].

Rarely circumscribed to the liver, the pathogenic burden of NAFLD is rather systemic. The excessive energy intake associated with the consumption of hypercaloric diets and the low energy requirements of sedentary lifestyles cause a homeostatic imbalance and the net retention of energy in the form of fat. This chronic metabolic imbalance underlies the development of obesity, insulin resistance (IR), Metabolic Syndrome (MetS), Type 2 Diabetes Mellitus (T2DM), and the ectopic accumulation of fat within the hepatic parenchyma [7–9]. Besides its shared aetiology, NAFLD presents a strong bidirectional relationship with MetS features and T2DM, exacerbating its severity and progression rate [10, 11].

Although Cardiovascular Diseases (CVDs) are the first cause of death in NAFLD patients, NASH significantly contributes to the mortality burden of cirrhosis and HCC, which combined account for 3.5% of deaths worldwide [2, 12, 13]. Population aging and the rising prevalence of T2DM, a risk factor for cirrhosis progression from NASH, predict an upcoming increase in liver-related mortality due to NAFLD [3] and envision NAFLD to become the leading cause of liver transplantation in Europe in the near future [14].

Despite the threat that NAFLD poses to human health, the public health response to NAFLD is straggling at multiple levels [15, 16]. NAFLD awareness amongst healthcare providers is low and adherence to NAFLD clinical guidelines regarding diagnosis, monitoring, and management is poor [17–20]. Similarly, NAFLD awareness amongst metabolic patients and the general population is scant [21–24] and therapeutic adherence amongst NAFLD patients is poor [25–27].

Whilst only 2% of the 5000 participants of a survey in New York City had heard of NAFLD, 17% of the 521 respondents to a telephone survey in Hong Kong had previously heard of NAFLD [21, 22]. Similarly, only 18% of 302 respondents in a study in Colorado had previously encountered the terms “Non-Alcoholic Fatty Liver Disease”, “NASH” or “fat in the liver” [23]. These studies suggest that NAFLD awareness is gained by interaction with healthcare providers [21, 23].

Lifestyle interventions addressing dietary regimes and physical activity patterns are the standard of care for NAFLD whilst constituting a crucial component of therapeutic strategies targeting other metabolic diseases [28, 29]. However, structured lifestyle programs to treat NAFLD are only provided in 7 European countries [30] and often report low success rates in promoting the weight loss necessary for an improvement in NASH histological features including fibrosis regression [29, 31–33]. Moreover, scarce motivation to adopt healthier lifestyles regarding nutrition and physical activity has been reported by a study exploring the readiness of NAFLD patients for behavioural changes [34]. Posited by models of health promotion, disease prevention and therapeutic adherence, the adoption of health behaviours is dependent upon disease awareness, disease-related practical skills, individual beliefs regarding disease and treatment, motivation and perceptions of self-efficacy [35–38]. Although several NAFLD-awareness campaigns are ongoing [30, 39, 40] and some lifestyle intervention studies currently include educational or psycho-behavioural components [32, 33, 41, 42], these seem largely designed from a top-down perspective. Yet, current models of health communication advocate tailoring the initiatives to the individual or community’s existing knowledge and capacities, ultimately providing the context-specific health knowledge and fostering the self-efficacy required to exert better control over their health [38, 43, 44].

Considering that the prevalence of NAFLD amongst T2DM patients is estimated to be as high as 70%, raising NAFLD awareness within this community seems of particular importance [45]. In this study, we intended to gain some insight into the NAFLD-related experiential and factual knowledge possessed by a group of T2DM patients and how that information may be used to make them more NAFLD-aware. Adding to the scarce literature in NAFLD awareness and complementing its focus on the quantification of pre-established theoretical constructs, the exploratory nature of qualitative research should contribute towards uncovering unforeseen ideas, understandings, opinions and motivations with potential to inform NAFLD public health interventions [46].

## Methods

Through a descriptive qualitative approach and under an interpretivist paradigm, this cross-sectional study sought to investigate T2DM patients’ awareness about NAFLD within the context of their T2DM illness and therapeutic experience [47]. For this purpose, semi-structured interviews were conducted at the Portuguese Diabetes Association (APDP). The development of the interview guide and the conduction of the interviews were performed by researchers with a biomedical background that facilitated content selection and the recognition and understanding of medical concepts shared by participants. Intending to challenge pre-established assumptions inherent within this biomedical perspective and trying to complement potential design and interpretation biases, the view of researchers with a background in psychology, sociology, patient communication and qualitative research were sought during the design and data analysis phases of the study.

### Research setting

The study was conducted at the Portuguese Diabetes Association (APDP), located in Lisbon (Portugal). Founded in 1926, APDP is the oldest diabetes association in the world. Initially conceived as a philanthropic organization seeking to provide free insulin to those who could not afford it, nowadays it is a modern health institution catering comprehensive diabetic care for over 30.000 members [48]. The APDP outpatient clinic is composed of a multidisciplinary team that provides integrated clinical care, serving as an ambulatory care facility for diabetic patients and as a referral site for complications management. Its approach is deeply invested in defending the rights of people with diabetes and their inclusion in society. Alongside medical services, APDP educational interventions strive towards patient empowerment by fostering self-management behaviours, discussing T2DM biomedical knowledge, glycaemic control, insulin therapy and foot care, as well as providing nutrition workshops, cooking classes, exercise training and workout sessions [49, 50]. There is no hepatology service at APDP and none of the education sessions are focused on NAFLD, thus providing the ideal setting of awareness for some aspects crucial for NAFLD without focusing on the disease itself.

### Development of the semi-structured interviews guide

The development of the semi-structured interview guide followed a sequential curation process. The content of the questions was decided according to the research aims and taking into account the available literature on NAFLD awareness consisting of quantitative studies employing the use of questionnaires and surveys to access large cohorts of participants [21–23]. The initial compendium for the interview included 18 questions regarding awareness of NAFLD as a diagnosis entity as well as epidemiology, aetiology, progression, prognosis, prevention and treatment of the disease, NAFLD risk perception in a T2DM context and general interest in NAFLD information.

Researchers MMR and SA, with a background in psychology and sociology, respectively, provided feedback on the interview guide draft based on their previous experience in qualitative research. Integrating their input, the topics of the interview were reformulated to better allow patients to share their experiences and reasoning and to facilitate a continuous flow of conversation. To that end and taking into account the expected low degree of NAFLD awareness, the first questions were dedicated to asking about participants’ knowledge about T2DM, with which they would be more familiar, rather than NAFLD. We believe this would set the context for exploring the relationship of participants with NAFLD-related topics. The interview guide included other topics that were then further elaborated by the participants, such as liver physiology, NAFLD pathophysiology and knowledge on dietary and exercise behaviours. This approach was substantially different from previous quantitative studies, which were designed from a health policy and management perspective, and thus probed awareness of NAFLD prevalence, diagnostic methods and clinical symptomatology. The content and structure of the interview guide were discussed between the research team, composed by psychologists, sociologists, biomedical researchers and a nurse practitioner to assure that it assessed relevant content regarding the study goal and that it was formulated to allow participants to express their views on the topics.

This version of the semi-structured interview consisted of 15 topics with probes enquiring about: a) T2DM disease knowledge and illness perceptions, b) knowledge on insulin resistance and the interplay between NAFLD and T2DM, d) liver function, fatty liver awareness, NAFLD disease knowledge on pathophysiology and progression of the disease, e) cirrhosis awareness and relationship with NAFLD, f) role of diet and exercise on T2DM and NAFLD therapeutic approaches and e) interest in receiving NAFLD education.

### Study population and data collection

From October to December 2018, 35 people attending consultation at the Portuguese Diabetes Association (APDP) in Lisbon were invited to participate in this study. A purposeful random sampling strategy on the basis of a diagnosis of either T2DM or pre-diabetes was used to select participants [51]. Recruitment was conducted in person at one of the waiting rooms of APDP headquarters. The waiting room manager provided an attendance list and appointed those patients with a longer waiting time for consultation. Those patients were sequentially and consecutively approached by researcher AMA and briefly introduced to the study. Participation was voluntary and involved face-to-face semi-structured interviews conducted in a private room at APDP by researcher MAP with AMA as an observer. MAP and AMA did not have contact with any of the participants prior to undertaking this study nor had any role in the clinical management of the interviewees.

The order and phrasing of the questions were adapted to each individual case, whilst probing and clarifying questions were used to facilitate information disclosure. The interview guide evolved through the process of data acquisition and analysis, enabling a better exploration of preestablished and emergent themes. The item of NAFLD surveillance behaviours was added after the first round of interviews and the item of interest in receiving NAFLD education was deemed redundant and dropped (the initial interview guide is available at Supplementary file 1).

The duration of the interviews was of approximately 30 min, which allowed for exploration of the topics of interest, including exemplifications by the patient and follow-up questions by the researchers. At the end of the interview, participants filled an anonymized demographics questionnaire that collected information regarding patient code, age, gender, level of education, occupation and time since T2DM diagnosis. The illustrative quotations in the results section are identified following (patient code, gender, age-range, education); the latter categorized in NO, no formal education; 1 °C, 1st cycle of primary school; 2 °C, 2nd cycle of primary school; 3 °C, 3rd cycle of primary education; SEC, secondary education; HI, higher education. The medical file of participants was not accessible. Except for T2DM status, all clinical information discussed here was obtained from patient recollections. All interviews were conducted in Portuguese and audiotaped with a voice recorder. Data collection was continued until thematic saturation was reached, a point in which the low expected incidence of new ideas in the data did not warrant further exploration [52]. Qualitative research expands the possibilities of addressing unpredicted topics and deeply understanding people’s choices and behaviours. At the same time, we acknowledge that the data collected is influenced by the research aims, the formulation style of the questions and the listening skills of the researcher conducting the interviews. The first round of interviews was immediately transcribed, evaluated and discussed by the multidisciplinary team in order to identify personal bias, correct the interviewer’s posture and minimize any form of unintended coercion on the interviews.

### Ethical considerations

This study was approved by the Ethics Committee for Health of APDP and written informed consent was obtained from all participants in the study before initiating the interviews. No compensation was provided to participants.

### Data analysis

All voice recordings were transcribed verbatim by a professional. The transcripts were read a first time for familiarization with the data and to check for transcription accuracy. A content analysis methodological approach guided the analytical process directed at unveiling the degree of NAFLD-awareness amongst T2DM patients, including their perceptions and factual biomedical knowledge on the disease [53]. The interviews were analysed using MAXQDA Standard 2018 (VERBI Software, 2017) [54]. The transcripts were imported to MAXQDA as soon as they were available and the process of data analysis was initiated concurrently with the last stages of data collection. Individual interviews were used as units of analysis, which was performed by the first author. The early transcripts served as a sample to systematically search and identify concepts of interest embedded in the participants’ responses to the open-ended questions of the interview process. Each of these concepts of interest was assigned a code. A preliminary coding scheme was developed based on the emerging patterns and repetitions found across transcripts. By fitting and grouping the coded concepts into pre-established themes, a final coding scheme was defined. All transcripts were sequentially analysed to fit the data into the following key themes: a) NAFLD-awareness, including aetiology and treatment, b) Progression to end-stage liver disease, c) Surveillance behaviours, d) T2DM Self-Management Knowledge and e) NAFLD risk perception. Using the strategy of persistent observation to ensure the credibility of the results, several consecutive analytical rounds were performed in each of the transcripts [55–57].

Researcher triangulation and audit trials were used to ensure rigor and analytic trustworthiness. The interview guide drafting process, the design of the study and the procedures used for recruitment, sampling, data collection and analysis were documented and stored. Alongside the first author as the main coder, the author MMR performed an independent analysis in 50% of all transcripts developing a parallel code book on the concepts of NAFLD-awareness, aetiology and treatment, as well as surveillance behaviours, which were provided to her as preestablished themes into which to fit the observed data. As a clinical psychologist, the expertise of MMR complemented that of the first author, trained in biomedical sciences. Used to calculate interrater reliability, the Average Kappa coefficient was of 8.89, suggesting strong agreement [58]. Later, the two authors (MAP and MMR) compared codes, discussed convergences and discrepancies and reached full consensus. The findings are reported below in anonymized quotes that were translated into English from the verbatim interview transcripts.

## Results

### Response rate and demographics

A total of 35 patients were approached to participate in this study whilst awaiting consultation. One patient refused to participate and one was not eligible for participation due to the lack of a T2DM diagnosis. The remaining 33 patients were interviewed between October 4th and December 7th, 2018. Three participants were excluded from the study after voluntarily interrupting the interview process, all but one out of concern of missing the medical appointment.

In the end, 30 of the T2DM patients approached were included in the study (attrition rate 14%). The median age of the participants was 65 years, 53% were female and 66% reported at least another case of T2DM in the family (Table 1). Of the 30 participants, 10 had been diagnosed with NAFLD in the past, including metabolic and drug-induced forms of NALFD. Considering that the highest incidence of T2DM is between the ages of 45 and 65 and that its prevalence increases with age, the epidemiological profile of this elderly cohort is similar to that of the global T2DM patient population and that of patients receiving care at APDP [59].

**Table 1:** Demographic profile

	N (%)
Gender	
Female	16 (53)
Male	14 (47)
Age (years), median (SD) (range)	65 (8.99) (50–87)
50–60 years	9 (30)
61–70 years	11 (37)
> 70 years	10 (33)
Education Attainment	
< 4 years (No Formal Education; NO)	1 (3)
4 years (1st Cycle Primary Education; 1 °C)	10 (33)
6 years (2nd Cycle Primary Education; 2 °C)	4 (13)
9 years (3rd Cycle Primary Education; 3 °C)	2 (7)
12 years (Secondary Education; SEC)	4 (13)
> 12 years (Higher Education; HI)	9 (30)
Employment status	
Employed	10 (33)
Unemployed	1 (3)
Retired	19 (63)
Type 2 Diabetes Mellitus (years), median (SD) (range)	
Time since diagnosis	17 (13.46) (0–48)

The illustrative quotations are identified following (patient code, gender, age, education); the latter categorized in NO, no formal education; 1 °C, 1st cycle of primary school; 2 °C, 2nd cycle of primary school; 3 °C, 3rd cycle of primary education; SEC, secondary education; HI, higher education

### NAFLD-awareness

#### NAFLD disease identity and causes

The term fatty liver was recognized by a total of 15 participants (Table 2). Of those, the majority were aware of the disease term because they had been diagnosed with NAFLD in the past or were suffering from NAFLD at the time. The rest had close relatives affected by the disease or gained awareness as a result of health information-seeking behaviours.

“At the level of the liver, it seems that it is not that bad, even though there is a bit of fat in the liver.” (#127.1, ♂, 61-70 years old, HI)

Infection by hepatitis virus, alcohol and non-alcoholic fatty liver disease, the latter encompassing both drug-induced NAFLD and metabolic NAFLD, were mentioned as the aetiological factors responsible for the development of fatty liver. Hypertriglyceridemia, hypercholesterolemia or the consumption of high-fat diets were the most commonly mentioned causes for metabolic NAFLD.

“I know that the fatty liver is from someone that has an excess of fat, or that has, eventually, higher levels of cholesterol. [...] But the, the fatty liver, in addition to being associated to, to an increase in volume, for some reason, I know it has to do with, with the liver not, not working so well in terms of the capacity of, of processing fats.” (#116, ♂, 61-70 years old, HI)

“The liver, look, when I had very high levels of triglycerides, the triglycerides attack the liver, I think. In the beginning they said that I drank a lot. I drink. Now I drink even less, but I was never an alcoholic … [...] Well, I have the liver, a bit fatty. Why, I don’t know, since the diet that I do is a moderate diet, I don’t eat fats, the food, when it goes to the pan, it already has no fats, and then, even in the plate, I even remove all the fats. I take [medication], that is to reduce my triglycerides. To clean up, clean up the fat from the blood, but I don’t eat fats. Who knows what this is.” (#106, ♂, >70 years old, 3°C)

#### Liver function and consequences of NAFLD

Knowledge about the liver and its physiological functions was scarce. About a third of the respondents perceived the liver as a filter for blood purification and 2 mentioned the production of bile but none recognized the role of the hepatic tissue in metabolism and energy homeostasis. There was a generalized lack of knowledge on the consequences of steatosis. A minority of patients mentioned hepatomegaly and the progressive loss of hepatic function after enquiring what happens in the liver when it becomes fatty.

“[The liver] is the one that produces … so, it purifies the blood, right? And then it even produces a substance that is also secreted into the food … and that helps to make the digestion, right?” (#132.1, ♀, 61-70 years old, SEC)

“Yes, about the liver, sometimes, the, in the morning, after a period of fasting a bit long, I have the diabetes high and they tell me it can be, it might have been low, but the liver, to compensate, started to, to strain for insulin, or to make the insulin go up. It’s not the insulin, sorry, yes … To rise the glycemia.” (#124, ♀, 61-70 years old, HI)

#### NAFLD treatment

Whilst dietary behaviours were often recognized as a component of NAFLD management actions, these participants seemed not to grant this lifestyle modification a therapeutic status. Exercise as part of the management strategy for NAFLD was mentioned by one participant. There was no reference to the beneficial effects of T2DM pharmacological agents on NAFLD, although some patients referred to anti-hypertriglyceridemic agents or antiviral medication as part of their fatty liver treatment.

“None. It is to eat, the more salads the better, and more stews and broils. It was what they ordered me to do. Now, medication I am not taking any.” (#105, ♀, 50-60 years old, 1°C)

“Ah, it is that same old conversation as always. It is the diet, it is the practice of exercise, it is taking care of the diet, it is not smoking, it is not drinking. It is all those things that are applicable to any little thing.” (#122.1, ♀, 50-60 years old, HI)

### Progression to end-stage liver disease

The concept of cirrhosis as a disease entity was recognized by the vast majority of participants. In most cases, patients identified the liver as the organ being affected and associated the disease with the excessive consumption of alcohol. Surprisingly, it was also recognized by more than half of the participants, that factors other than alcohol, in most cases reported as unknown, could also cause cirrhosis.

“Yes, the cirrhosis is in the liver, but I don’t drink. They say that it is from drinking, it gives cirrhosis. [ … ] The liver melts all down. The liver doesn’t … , starts to bloat a lot. There is a guy next door, that, that one drinks Whiskey and, and such, he has been hospitalized a few times, he is about to die, but then he comes back. He bloats. He is slim, but he has a giant belly. It is the liver, the liver makes it bloat, it starts to be, and laying down, he removes, he had to remove liquids … because he is … , he is sick. It is because of the alcohol.” (#119, ♀, 61-70 years old, 1°C)

“And there are people that has cirrhosis and that never drank. Did you know? I think you know. And I also cannot, cannot explain why, because, usually, between people that drink, that drink a lot have cirrhosis. But there are people that never drank and already had cirrhosis and died of cirrhosis.” (#101, ♂, 50-60 years old, HI)

Showing a clear lack of awareness on the progression of NAFLD towards late-stage liver disease, only 3 patients reported a clear association between fatty liver and cirrhosis. Other identified causes of cirrhosis included viral infection, medical drugs and T2DM. Awareness of cirrhosis being a life-threatening condition was prevalent, as mentioned by a total of 12 respondents.

“I think that [cirrhosis] is, is what you call a very advanced state of fatty liver, isn’t it? And the liver does no longer work, or will work very badly when it reaches that state, isn’t it?” (#109.1, ♂, 50-60 years old, HI)

“Cirrhosis yes, that is already a disease that, a liver disease, isn’t it? And in the past, associated with alcoholic beverages, but this doesn’t need to be the case. Associated with alcoholic drinks, isn’t it? It can be associated with diabetes. [ … ] Yes, but this, because the blood that transports the fats, the sugars and who knows what more, ends up tiring the liver, no? With so much, with so much stuff, maybe.” (#132.2, ♀, 61-70 years old, SEC)

The interview guidelines did not include any explicit enquiries regarding Hepatocellular Carcinoma (HCC) and no unprompted mentions by the participants were observed in this study. Conceptual representations on the effects of cirrhosis on the morphology of the liver were diverse, referring to its dilation or swelling, degradation, melting down, burning out, destruction, or the state in which the liver stops working or starts dying. This knowledge seems to be mostly acquired through the illness experience of members of their social networks.

### T2DM self-management knowledge

Knowledge of the multifactorial nature of T2DM treatment was widespread. Regarding diet composition, avoidance of either simple or complex sugars was ubiquitously referred. Whilst 9 participants reported the need to avoid simple sugars, 4 indicated the need to reduce consumption of complex carbohydrates and 15 the need to cut on both. Portion size reduction as a strategy for caloric restriction was frequently mentioned. Avoidance of fats in the diet was reported by two-thirds of the participants. A total of 24 patients were aware of the hyperglycaemic impact of food consumption. Of these, 14 focused on the role of carbohydrates in the diet, 7 patients reporting the hyperglycaemic influence of both starches and simple sugars and the rest only referring to the latter. Seeming to stem from experience rather than medical instruction, the hyperglycaemic effect of lipid intake was mentioned by 6 participants.

“Yes, salads, we can eat everything, but in very little quantities. For example, boiled potatoes, these need to be two small potatoes, of the size of an egg. Then, I can eat all the vegetables that I want, greens, broccoli, cauliflower, as a sider to that food to fill up more the stomach, that’s it. Because that does not make “the diabetes” go up. And if we were to eat a lot of farinaceous foods, “the diabetes” goes up later, isn’t it? I can’t eat a lot of bread, isn’t it? It also makes them go up. If I eat a lot of fruit, they also go up. We can only eat two pieces of fruit a day and not so sweet. I know all of this, so be it that sometimes one flees. [ … ] More fish than meat because it is healthier, isn’t it? In all ways. They don’t have that much stuff for them to rise up. [And fats?] Yes, nothing of fats, just white meats. Rabbit, chicken, just white meats like this. Cow meat and such, are not recommended for us to eat, that’s it. Because it is fattier. That’s it. It’s more, they recommend more grilled and boiled meals." (#110, ♀, 50-60 years old, 1°C)

About half of the participants in this study report having a clear structure for regular exercise practice, mostly aerobic activity. The rest mentioned occasional walks, work-related physical activity or home duties as part of their activity. A total of 18 participants acknowledged the effect of exercise practice on glycaemic control.

“People end up much more at ease. If I were to have “the diabetes” a bit higher, I don’t mean that I was to have, 200 and some like I have now, but if I were to have 180 and I would want to lower it down, for example, if I were to go do that exercise of the Free Waters Aqueduct and come back, [ … ] and when I go to measure, many times I have 140. I mean, I guess it is from, from the exercise, that in the meantime, it made me lower it down.” (#112, ♀, 61-70 years old, 2°C)

### NAFLD risk perception

Crucial to understanding the relationship between T2DM and NAFLD, the term insulin resistance was unknown to all study participants. Nonetheless, some patients understood and elaborated on concepts related to IR. Two patients described the process of gluconeogenesis as the responsible mechanism for their fasting hyperglycaemia, although only one of them attributed it to the liver (quote from #124 above). When enquiring about the connection between the liver or NAFLD and T2DM, responses were diverse. Two participants considered that T2DM and NAFLD share an underlying cause whilst two others, explicitly reporting to be diagnosed with NAFLD prior to the development of T2DM, did not consider any aetiological relationship between the two conditions.

“Yes. The fatty liver happens through the excessive accumulation of fat in the liver, isn’t it? Therefore, and it makes it not work well, and alters some tests, that are values of an analysis that, that are made, from which you know if there is fatty liver or not, I think, isn’t it? [ … ] Why is it that it accumulates there, I don’t know...Probably because, I don’t know, since that is a little filter of the blood, isn’t it? The blood controls that whole thing, so the liver is there, if the blood is not in good conditions, probably it is all retained there, isn’t it? [ … ] I don’t associate much the situation of the fatty liver with the insufficiency of the functioning of the pancreas. I don’t know. But it will have more to do with other factors, more diet-related.” (#109.2, ♂, 50-60 years old, HI)

“Yes. Ah, what is it that happens? Well, poor things, our liver and our pancreas get tired and start to say that they need a, a different kind of diet and a different type of life. If people don’t do it, it [interjection] it stops producing insulin. [ … ] The liver can also be related with diabetes, yes sir, because the liver, then, we don’t eliminate that what the liver puts out there, it also helps all the rest, all the rest helps the diabetes. This is all, all an endless cycle. One thing leads to the other, the other leads to that other, the other to the next, and the liver is in the middle of all that.” (#122.2, ♀, 50-60 years old, HI)

One participant considered NAFLD to be a risk factor for the development of T2DM.

“My son, for example, he doesn’t have diabetes, but has fatty liver. [ … ] Listen, it’s like this. And that’s it. But since he has fatty liver … , he could have diabetes, isn’t it? Maybe he has more … things to have diabetes. [ … ] Already my niece, the daughter of my sister [besides T2DM] also has fatty liver. But she is all fatty, all of her.” (#111, ♀, >70 years old, 1°C)

Conversely, 7 patients conceived liver damage as a consequence of T2DM. Of these, three were NAFLD aware and four were NAFLD unaware. Interestingly, one of the NAFLD unaware patients reported T2DM as a risk factor for the development of cirrhosis (quote #132.2 above).

“Yes, yes. So, the sugar is not absorbed or annulled, let's call it this way, and it is hosted in other organs, the liver, the kidneys, the sight, out there, damaging them. [...] The fact that the liver has to work more or, like, the sugar be, being more sugar than the liver can process, or that has to analyse, causes that it becomes not as healthy.” (#121.2, ♀, 50-60 years old, HI)

### NAFLD surveillance behaviours

A total of 10 participants reported having had an abdominal echography in the past as part of surveillance behaviours to control the function of several organs, including the liver. As mentioned above, NAFLD-awareness seems to be associated with its diagnosis. Only one of the NAFLD aware patients who did not have a NAFLD diagnosis reported engaging in periodical echography screenings. From those reporting a NAFLD diagnosis, half did not perform any post-diagnosis follow-up exam to verify the state of the liver and the possible progression of NAFLD along its continuum.

“The nephrologist told me: “It is not very worrying, but I also noticed in the analysis that I requested that, besides the, the, the small deviation in the kidney, there is also something here in the liver, I suggest you get that checked”. But in reality, I never again...went to see that through, no.” (#127.2, ♂, 61-70 years old, HI)

“Yes. It is the fatty liver. I also, at the time, I made echography scans, indeed, I had fatty liver. After that, I think I never again did any. That means, I don’t know if it went down, if it still [laughs] has … But yes, I already had the liver affected.” (#121.1, ♀, 50-60 years old, HI)

**Table 2:** Prevalence of NAFLD awareness and NAFLD-related beliefs

	N	% NAFLD-Aware	% Total
**NAFLD awareness**			
**NAFLD as disease entity**			
NAFLD awareness	15	100	50
History of NAFLD	10	66.67	33.33
**NAFLD causes**			
High-Fat diets, hypercholesterolemia or hypertriglyceridemia	9	60.00	30.00
Alcohol	7	46.67	23.33
Hepatitis virus	2	13.33	6.67
Pharmacological drugs	2	13.33	6.67
**Liver function**			
**‟**Filter for blood purification”	9		30.00
Secretion of bile for digestion	2		6.67
Metabolism / Energy homeostasis	0		0.00
**NAFLD treatment**			
Dietary regimes	6	40.00	20.00
Physical activity or exercise regimes	1	6.67	3.33
**Progression to end-stage liver disease**			
**Cirrhosis as disease entity**			
Cirrhosis awareness	27		90.00
Liver as affected organ	24		80.00
Life-threatening status	12		40.00
Awareness from vicarious experience	14		46.67
**Cirrhosis causes**			
Alcohol	24		80.00
Non-identified factor	9		30.00
Fatty Liver	3		10.00
T2DM	1		3.33
Hepatitis Virus	1		3.33
Pharmacological drugs	3		10.00
**NAFLD risk perception**			
Insulin resistance awareness	0		0.00
**Relationship between liver or NAFLD and T2DM**			
NAFLD and T2DM - shared underlying cause	2		6.67
NAFLD and T2DM - no etiological relationship	2		6.67
NAFLD as a risk factr for T2DM	1		3.33
T2DM as a risk factor for liver damage	7		23.33
**NAFLD surveillance behaviors**			
NAFLD-aware with prior diagnosis	5		16.67
NAFLD-aware with no prior diagnosis	1		3.33
**T2DM self-management knowledge**			
**Dietary knowledge**			
Hyperglycemic effect of food intake	24		80.00
Avoidance or reduction of both simple and complex carbohydrates	15		50.00
Glycemic impact of simple and complex carbohydrates	7		23.33
Avoidance or reduction of simple sugars	9		30.00
Glycemic impact of simple sugars	7		23.33
Avoidance or reduction of complex sugars	4		13.33
Glycemic impact of complex sugars	0		0.00
Avoidance of fat	20		66.67
Glycemic impact of fat consumption	6		20.00
Portion size reduction (caloric restriction)	18		60.00
**Exercise knowledge**			
Hypoglycemic effect of exercise practice	18		60.00

*Abbreviations*: *NAFLD* Non-Alcoholic Fatty Liver Disease, *T2DM* Type 2 Diabetes Mellitus

## Discussion

To our knowledge, this is the first qualitative study to assess NAFLD-awareness in a T2DM cohort and report their knowledge regarding aetiology, pathophysiology, treatment and disease progression of NAFLD, as well as its relationship with T2DM. In this study, whilst half of the participants recognized the concept of fatty liver, awareness on NAFLD as a disease entity was only reported by less than one-third of the participants, and seemed to be associated with its diagnosis. Indeed, in previous studies, NAFLD-aware patients were more likely to be high-risk metabolic patients, to have a history of liver disease or have undergone prior liver function tests and have attended T2DM education sessions [21, 23]. Higher than the percentage of NAFLD aware patients in previous studies, this might be the result of the high prevalence of NAFLD amongst T2DM patients [11, 21, 23].

Nonetheless, knowledge on the aetiology, pathophysiology and treatment of NAFLD was very scarce. Similar to the results of previous studies, viral infection, alcohol and medical drugs were mentioned as causes of fatty liver, whilst those referring to the aetiology of NAFLD unequivocally cited fat metabolism, referring to either hypertriglyceridemia, hypercholesterolemia or the consumption of high-fat diets [60]. None of the participants referred to physical inactivity or heritability as aetiological factors for NAFLD. The trend of undermining the role of physical activity in the aetiology of NAFLD was also reflected when discussing therapeutic strategies for this liver disease. These results also seem to support findings of previous studies suggesting a lack of perceived benefits of exercise practice amongst NAFLD patients, perceptions that were thought to be responsible for a lack of adherence to physical activity as preventive or therapeutic behaviour [34, 60, 61]. These knowledge gaps, together with a generalized tendency to undermine the therapeutic status of dietary and physical activity regimes, could be conductive of inadvertent non-adherence.

Alongside the low rates of NAFLD awareness, the poor understanding of liver physiology and fatty liver pathophysiology might be precluding the formation of beliefs and attitudes conductive of preventive behaviours. The most common conceptualization of hepatic function devised the liver as a blood filter and conceived a fatty liver as the state resulting from the retention of fats following the depuration of fatty blood attributed to hypertriglyceridemia, hypercholesterolemia or the consumption of fatty diets. Nonetheless, our data portrays a generalized lack of knowledge on the metabolic role of the liver. Moreover, being misinformed about the pathological consequences of steatosis was prevalent.

Unlike NAFLD, awareness of the concept of cirrhosis as a disease entity was widely observed throughout our sample. Almost ubiquitously recognized as a liver pathology, excessive alcohol consumption was described as the main aetiological factor for the disease and invariably linked to cirrhosis awareness. Such knowledge seemed to be learned vicariously, through the life experiences of family members and friends. Less common, but still prevalent was the notion that cirrhosis is a life-threatening condition and that other factors besides alcohol can also cause this end-stage liver disease. Nonetheless, the nature of the non-alcoholic aetiological factors reported was unknown in most cases, only a minority recognizing NAFLD as a risk factor for the development of cirrhosis.

Our findings underline a knowledge gap on the progression from non-alcoholic steatosis to end-stage liver disease in this cohort. Moreover, the reported low rates of participation in both preventive and surveillance screenings, as well as the low level of concern in the narratives regarding hepatic steatosis, especially those portrayed by patients not engaging in such programs, suggests a lack of perceived severity of the health implications of a fatty liver diagnosis, corroborating previous findings [24].

The detrimental potential of beliefs of low perceived severity on the likelihood to take preventive action could be further exacerbated by a lack of knowledge on the increased susceptibility of T2DM patients in developing NAFLD. Low NAFLD-awareness amongst T2DM is particularly concerning due to the high prevalence of NAFLD amongst this metabolic group and the bidirectional synergistic interplay of these disorders. On one hand, T2DM exacerbates the natural course of NAFLD, promoting the development of NASH, fibrosis and end-stage liver disease, including cirrhosis and HCC. On the other hand, the presence of NAFLD as comorbidity seems to be associated with a worse metabolic profile, the need for higher insulin doses and the severity of diabetic microvascular complications in T2DM patients. Furthermore, those individuals with both T2DM and NAFLD are at a higher risk of CVDs and report worse Health-Related Quality of Life (HRQoL) [62–64].

The disassociation between the metabolic conditions seemed apparent when discussing the pathophysiology of these diseases. The patient’s narratives depicted NAFLD as a fat-centric pathology driven by a disruption of fat metabolic fluxes whilst T2DM was conceived as a sugar-centric condition. The documented knowledge gap on the concept of IR, the underlying aetiological mechanism driving the pathophysiology of both NAFLD and T2DM, could further explain this disassociation. These results contrast those of previous quantitative studies, which perhaps due to the guiding nature of their multiple-choice questions, reported a moderate awareness of the concept of IR and the relationship of NAFLD with other metabolic disorders [21, 22].

Interestingly, our findings suggest that perceptions around the relationship between NAFLD and T2DM were constructed through personal and vicarious experiences and depended upon the temporal sequence of diagnosis. Patients who had a NAFLD diagnosis prior to T2DM established no connection between the two metabolic disorders. Conversely, a diagnosis of NAFLD as a T2DM patient seemed to lead to the belief that this hepatic condition was a consequence of diabetes comparable to T2DM macro- and micro-vesicular consequences such as nephropathy, neuropathy or retinopathy [65]. Such a conclusion seems further supported by the fact that the liver was mentioned as an organ affected by T2DM regardless of NAFLD-awareness.

The low rates of NAFLD knowledge in this and other studies suggest that a majority of T2DM patients, high-risk metabolic patients and the general population would benefit most from interventions that raise awareness on the disease, thus facilitating the transition from an unawareness state to proactive stages of covert or even overt behaviour [37, 66].

Likely, stressing factual knowledge able to influence the beliefs underlying behavioural intention might induce unengaged NAFLD aware individuals to contemplate taking precautionary action against NAFLD incidence or progression. Indeed, the NAFLD-aware participants in our study displayed low levels of threat perception regarding NAFLD. It would thus be expected, that increasing knowledge in NAFLD progression to the well-known life-threatening condition of cirrhosis would increase the perceived level of severity attributed to NAFLD as a disease entity.

Likewise, facilitating awareness on the concept of IR and the increased risk of T2DM patients regarding the incidence and severity of NAFLD could influence beliefs of perceived susceptibility to the disease. As predicted by the Health Belief Model (HBM), such increased awareness, together with information highlighting the underlying effects of healthy diets and physical activity regimes driving the benefits of endorsing such health behaviours should prompt the adoption of preventive and therapeutic behaviours [35]. Increasing NAFLD awareness amongst T2DM patients might increase participation in screening and surveillance programs for liver disease, facilitating primary and secondary prevention, but also boost the motivation to adhere to the lifestyle intervention component of T2DM treatment.

Although this rationale remains to be tested through intervention studies, research on the factors influencing adherence reveals that especially for lifestyle interventions, lack of information and self-management skills, self-efficacy issues and lack of motivation are amongst the major documented barriers for adherence [36, 67]. NAFLD perceived severity, disease knowledge and self-efficacy seem to be correlated with better adherence to dietary recommendations [60] whilst the level of concern of dying due to HCC, self-confidence in HCC knowledge and the patients’ degree of participation in decision-making processes of their disease management have been reported as predictors of utilization of HCC surveillance services [68].

To strategically maximize its impact on factual knowledge acquisition but also motivation and behavioural skills, NAFLD health communication initiatives should be designed to cater to the information needs and skills of individuals and communities. For this population, the construction of NAFLD knowledge around the cultural model for liver physiology as a blood filter as well as the shared explanatory models and popular theories used to explain cirrhosis and fatty liver could be of benefit [69–72].

Moreover, considering that the therapeutic plan for NAFLD management is embedded within the treatment approach for T2DM [73], diabetic patients with high T2DM-awareness and self-management skills possess privileged knowledge on NAFLD-relevant dietary and exercise behaviours as compared to other non-diabetic NAFLD patients. Yet, as reflected by this study, not all patients possessed the same degree of T2DM self-management knowledge [74–76]. Further research in this direction might support the inclusion of NAFLD-awareness material in T2DM self-management programs to be conceived around the temporal dimension of T2DM tacit knowledge acquisition, tailoring its content according to their level of T2DM self-management knowledge.

### Strengths and limitations

This is to our knowledge the first qualitative study addressing the topic of NAFLD awareness. Although innovative, this study has several limitations. The single-center study design and the small population cohort, restricted to older individuals of Portuguese nationality with low levels of formal education, limits the generalizability, transferability and external validity of our findings. Moreover, the adscription to confidentiality protocols at the Portuguese Diabetes Association precluded member checking as a technique to further ensure the credibility and trustworthiness of the data. Additionally, the lack of access to clinical data negated correlation analysis between awareness and clinical outcomes.

Our aim was not to infer the knowledge and perceptions at a population level, more representative of quantitative approaches already present in the literature, but to grasp a better understanding of what NAFLD means from first-hand experience and how this meaning is derived, built and modelled by experience with T2DM. The findings of this study can guide future research informing the development of NAFLD-related messages for public health interventions, especially those that seek to extend awareness and participation of most vulnerable groups in health promotion. In this study, over 56% of the participants had not attained secondary education. Low formal education is associated with low health literacy and worse health outcomes. These populations are the most burdened by chronic illnesses and the hardest to engage in adherence and prevention programs [71, 77–80]. Moreover, not only do the participants of this study represent a very small portion of total consultations at APDP, but they also differ considerably on their degree of experience with the disease, as their T2DM history ranges from 0 to 48 years. Illness experience is related with beliefs of self-efficacy and self-management knowledge, which in turn can shape the information needs of these patients in regards to NAFLD [76]. Future studies should address this issue.

By better addressing the needs of these communities, planning and implementation of public health interventions would be expected to increase the capacity of these individuals to freely exert their health citizenship. Future qualitative research exploring the construct of NAFLD awareness with other populations and communities, exploring the relationship between NAFLD knowledge and health-related behaviours, or querying for additional constructs such as HCC, one of the main end-stage liver disease outcomes of NAFLD would likely enhance the collective capacity to respond to this public health threat.

## Conclusions

NAFLD represents one of the most pressing public health threats today and awareness of this disease entity was low amongst the participants in this study, which as T2DM patients are at a higher risk of suffering from this chronic disease. Although the construct of cirrhosis was familiar to the majority of respondents, knowledge on the relationship between NAFLD, T2DM and end-stage liver disease was low. Unmet information needs under the theoretical construct of NAFLD-awareness hinder not only NAFLD diagnosis and management at an organizational level but might also be precluding metabolic patients from actively participating in the decisions concerning their health, including adherence to treatment and surveillance. The information gathered in these qualitative research studies can be used as input for the development of tailored health communication initiatives and self-management programs ultimately facilitating adherence to treatment as well as primary and secondary NAFLD prevention.

## Supplementary information

**Additional file 1** Semi-structured interview guide

**Additional file 2.** SRQR Checklist

## Data Availability

The datasets used and/or analysed during the current study available from the corresponding author on reasonable request.

## Abbreviations

APDP: Portuguese Diabetes Association
CVDs: Cardiovascular Diseases
HBM: Health Belief Model
HCC: Hepatocellular Carcinoma
HRQoL: Health-Related Quality of Life
IR: Insulin Resistance
MetS: Metabolic Syndrome
NAFLD: Non-Alcoholic Fatty Liver Disease
PAPM: Precaution Adoption Prevention Model
T2DM: Type 2 Diabetes Mellitus
