# Supplementary material for: Insights from qualitative research on NAFLD awareness with a cohort of T2DM patients: time to go public with insulin resistance?
Source: BMC Public Health. 2020 Jul 20;20:1142. doi: 10.1186/s12889-020-09249-5 (PMC7372774; doi:10.1186/s12889-020-09249-5)
Supplement: Supplementary file 1 — Additional file 1 Semi-structured interview guide [file 12889_2020_9249_MOESM1_ESM.docx]

# Semi-structured interview guide

1. What do you know about Type 2 Diabetes? Can you explain how it evolves?
2. What do you know about insulin? And insulin resistance?
3. Do you think that having Diabetes, affects your risk to develop fatty liver? Why?
4. What do you know about the function of the liver?
5. What do you know about the fatty liver? (Non-Alcoholic Fatty Liver: NAFLD)
6. How does one get a fatty liver?
7. How does the fat accumulate in the liver? Where does the fat come from?
8. What happens to the liver as it accumulates fat? How does the disease of the fatty liver progress?
9. What do you know about hepatic cirrhosis? Do you think it’s related with fatty liver?
10. What do you think is the treatment for a fatty liver?
11. What do we have to eat or avoid eating to have a healthy diet?
12. How does exercise practice help towards the maintenance of a healthy lifestyle?
13. By whom and how would you like to receive this information about the fatty liver and the influence of healthy lifestyles?
