# Supplementary material for: Insights from qualitative research on NAFLD awareness with a cohort of T2DM patients: time to go public with insulin resistance?
Source: BMC Public Health. 2020 Jul 20;20:1142. doi: 10.1186/s12889-020-09249-5 (PMC7372774; doi:10.1186/s12889-020-09249-5)
Supplement: Supplementary file 2 — Additional file 2. SRQR Checklist [file 12889_2020_9249_MOESM2_ESM.docx]

**Standards for Reporting Qualitative Research (SRQR)**

[http://www.equator-network.org/](http://www.equator-network.org/)

**CHECKLIST**

| **No.** | **Topic** | **Item** |
| --- | --- | --- |
|  | **Title and abstract** |  |
| S1 | Title | Reported (study as qualitative)  (Cover section, P11, L5) |
| S2 | Abstract | Reported (sections)  (Abstract section, P15, L7, L19, L24 and L34 for Background, Methods, Results and Conclusion sections respectively) |
|  | **Introduction** |  |
| S3 | Problem formulation | Reported (NAFLD as PH threat)  (Background section, P16, L8-10) |
| S4 | Purpose or research question | Reported  (Background section, P17, L51-57) |
|  | **Methods** |  |
| S5 | Qualitative approach and research paradigm | Reported (descriptive, interpretivist)  (Methods section, P18, L14-18) |
| S6 | Researcher characteristics and reflexivity | Reported (qualifications/experience, relationship with participants)  (Methods section, P18, L20-34; P19, L54-58 and P20, L4-7; P20, L36-44; P21, L30-40) |
| S7 | Context | Reported (research setting)  (Methods section, P18, L38-50 and P19 L4-9) |
| S8 | Sampling strategy | Reported (purposeful random, thematic saturation)  (Methods section, P20, L26-30 and P21, L24-28) |
| S9 | Ethical issues pertaining to human subjects | Reported (ethic’s approval, participant consent)  (Methods section, P21, L44-49) |
| S10 | Data collection methods | Reported (interviews, voice record, dates, data collection process) (Methods section, P20, L24-40; P21, L4-12 and L22-28) |
| S11 | Data collection instruments and technologies | Reported (questionnaires, audio recorders)  (Methods section, P20 L26-27; P21, L22-28) |
| S12 | Units of study | Reported (individuals, nº, demographic characteristics)  (Methods section, P22, L10-12; P21, L8-19) |
| S13 | Data processing | Reported (transcription, data entry, verification, coding)  (Methods section, P21, L53-60; P22, L4-32) |
| S14 | Data analysis | Reported (software, fit of data into themes, involved researchers) (Methods section P22, L4-56) |
| S15 | Techniques to ensure trustworthiness | Reported (triangulation, audit trial)  (Methods section, P22, L33-56) |
|  | **Results/Findings** |  |
| S16 | Synthesis and interpretation | Reported (main findings, quantifications)  (Results section, P23-P33 and Table 2) |
| S17 | Links to empirical data | Reported (quotes as evidence)  (Results section, P23, L44-47; P24, L4-34 and L42-55; P25, L16-25 and L40-59; P26, L13-26; P27, L9-27 and L36-44; P28 L10-53; P29, L4-12 and L30-43) |
|  | **Discussion** |  |
| S18 | Integration with prior work, implications, transferability, and contribution(s) to the field | Reported (integration with prior research and scholarship)  (Discussion section, P31-33) |
| S19 | Limitations | Reported (Strengths and Limitations section, P34 and P35) |
|  | **Other** |  |
| S20 | Conflicts of interest | Reported (None) (Cover section, P13, L31-34) |
| S21 | Funding | Reported (Cover section, P13, L37-50) |
